# The impact of a nutrition course on self-epistemic authority, professional meaning as mediating factors on professional identity among nursing students

**DOI:** 10.1186/s12912-024-02220-4

**Published:** 2024-08-12

**Authors:** Miriam Theilla, Dorit Weil-Lotan

**Affiliations:** Nursing Department, Tel Aviv-Yaffo Academic College School for Nursing Sciences, Hever Haleumim 10, Yaffo -Tel Aviv, Israel

**Keywords:** Professional identity, Self-epistemic authority, Professional autonomy, Nutrition, Nursing students

## Abstract

**Background:**

The professional competence of nursing personnel is integral to the efficacy of nursing procedures. Educational endeavors, especially those encompassing professional training programs, are critical in fostering a professional identity among nurses. The role of nurses within a multi-disciplinary nutrition team has the potential to enhance professional identity and improve the quality of care provided.

**Objective:**

This study aimed to explore the potential impact of knowledge acquisition and practical nutrition education on the development of professional identity among nursing school students. Furthermore, we hypothesize that professional autonomy and self-epistemic authority mediated the relationship between a sense of meaning, professional mission, and professional identity.

**Design:**

A cross-sectional survey compared nursing students who had completed a practical nutrition course with those who had not. The study measured professional identity, professional autonomy, self-epistemic authority, and sense of meaning. Data collection was conducted using validated questionnaires, with questions tailored to suit the study demographic. Mediation analysis was conducted on the combined sample of both groups.

**Participants:**

The study included 98 nursing students, divided into a study group (57 students who completed a nutrition course) and a control group (41 students who did not complete the course).

**Results:**

Significant differences were found between the groups in measures of professional identity (t = 3.42, *p* < .001), professional autonomy (t = 2.93, *p* < .005), and self-epistemic authority (t = 2.78, *p* < .007). There was no significant difference in the sense of meaning (t = 1.45, *p* = .150). Mediation analysis on the combined sample revealed that self-epistemic authority mediated the relationship between professional meaning and professional identity, while professional autonomy did not.

**Conclusion:**

The findings suggest that practical nutrition education enhances nursing students’ professional identity, autonomy, and self-epistemic authority. Future studies should include larger and more diverse samples to further explore these relationships.

## Introduction

Nurses possessing a strong professional identity and awareness of their unique contributions to the healthcare system play a crucial role in acknowledging the importance of professional identity [1]. Professional identity is defined as the self-perception of nurses within their occupational realm. It represents a critical element across professions, with the nursing sector actively engaging in dialogues to affirm its status as an independent discipline.

Nursing is an old profession but relatively new in the academic field. It became an academic profession at the end of the twentieth century and the beginning of the twenty-first century [2]. There is a clear link between nursing as a scholarly profession and developing one’s professional identity. According to Findlow (2012) [3], establishing a nurse’s professional identity becomes challenging when academic subjects prioritize nursing values over those of academia. In New Zealand, a study conducted among nurse teachers [4]. revealed that academia plays a vital role in the professional discipline of nursing and the cultivation of their professional identity. Researchers have additionally endeavored to ascertain whether nursing qualifies as an academic profession [5]. Despite a consensus among nursing professionals regarding their fundamental duty to deliver care, a disconnect persists between the theoretical principles and their practical implementation [6].

The significance of appropriate professional education and clinical experience as a contributory factor to personal fulfillment within the nursing role is widely recognized [7].

A qualitative study revealed that the perceptions of nursing students regarding the knowledge gained through their academic journey, encompassing clinical training, are instrumental in the shaping of their professional identity [8].

A pivotal role of education is cultivating self- epistemic authority among students. The student’s perception of how their instructors receive nutritional knowledge and treatment advice significantly affects their sense of self -epistemic authority [9] In the context of professional knowledge, self-epistemic authority pertains to the degree of trustworthiness and credibility attributed to an individual’s expertise and knowledge within a particular profession or discipline [10]. Thus, it becomes apparent that the cultivation of specialized clinical knowledge areas, such as nutrition, is crucial among nursing personnel. Nurses serve as integral components of multidisciplinary teams in nutrition care. Providing nurses with both the knowledge and practical tools in this domain not only enhances their self-epistemic authority but also promotes cognitive adaptability. This quality is vital for the identification and critical assessment of their knowledge and convictions [11]. Furthermore, such empowerment supports nurses in executing informed and independent professional judgments, free from external influences [12].

Within the domain of nursing, professional autonomy is characterized by the authority to make decisions and the liberty to act predicated upon the professional knowledge base dedicated to human care [13]. Research within the medical field has illuminated the pivotal role that professional knowledge plays in promoting professional autonomy and nurturing professional identity across a spectrum of diverse disciplines [14].

This study investigated the potential impact of acquiring knowledge and receiving practical nutrition education on developing professional identity among nursing school students. Additionally, we hypothesize that professional autonomy and self-epistemic authority mediated the relationship between a sense of meaning, professional mission, and professional identity.

## Methods

### Research design and setting

This study employed a cross-sectional survey methodology, utilizing a convenience sample comprising 98 Nursing students drawn from various academic years at a prominent college located in central of the country. Recruitment and data were collected from December 2022 to September 2023. Data collection was facilitated via a structured questionnaire administered to participants who had either completed a nutrition course (*n* = 57) or had not yet enrolled in or completed such a course (*n* = 41).

The Health Promotion course in nutrition is a newly added course that is offered in the later years of study (semesters 6–7/8). The primary goal of this course is to build upon the knowledge gained in previous classes and apply it to specific populations. We built the learning outcomes including assignments according to Bloom’s taxonomy [15]. students in this course are expected to move beyond simply acquiring knowledge and understanding. They are encouraged to analyze, synthesize, and evaluate the course material from a clinical perspective. This higher level of critical thinking enables students to explore concepts such as competence and self-epistemic authority, which play a crucial role in shaping their professional identity as nurses.

### Participants

All nursing students enrolled at the college were invited to partake in the research, with 120 students consenting and volunteering to participate. However, 22 of these volunteers failed to complete the majority of the questionnaire items, resulting in the exclusion of their responses from the analysis. Of the 98 students who were included in the analyses, 28 were first-year students (28.6%), 38 were second-year students (38.8%), 11 were third-year students (11.2%), and 21 were fourth-year students (21.4%).

### Measures

#### Demographic questionnaire

This questionnaire comprised six questions designed to gather data on participants’ age, gender, religion, marital status, academic year, and whether they had participated in and completed a nutrition course.

#### Professional identity

The 41-item scale developed by Fisherman & Weiss (2011) [16]. for application among schoolteachers and subsequently modified for this study to suit the nursing profession probes into four different dimensions of professional identity. These dimensions include ‘confidence in the professional choice’, exemplified by statements such as, “I am certain choosing to become a nurse was the correct decision”, ‘feeling of self-efficacy’, with examples such as, “I believe I can be an effective nurse”, ‘sense of mission’, illustrated by, “I view the nursing profession as a calling”, and ‘reputation of education and teaching’, represented by sentiments such as, “when someone shows disrespect towards nurses, I take it personally.” Participants were instructed to assess the extent to which each statement accurately reflected their experience, using a 4-point Likert scale ranging from 1 (completely untrue) to 4 (completely true). The overall score for professional identity was derived by computing the mean across all 41 items ranging between 1 (low level of professional identity) and 4 (high level of professional identity). The reliability coefficient (Cronbach’s alpha) for this scale in the present study was 0.95.

#### Professional Autonomy

The scale comprises seven questions grounded in the perceived autonomy support construct by Grolnick et al. (1991) [17], initially conceptualized within the context of children’s perception of parental support and subsequently modified for applicability to the present cohort. These questions explore the degree of professional autonomy perceived in the workplace, with items such as, “my opinions are acknowledged and valued at work”, “my colleagues are generally willing to consider my perspectives and insights on nutritional issues”, and “I have the autonomy to implement my decisions in the workplace”. Participants were prompted to evaluate how accurately each statement reflects their feelings using a 5-point Likert scale, ranging from 1 (completely untrue) to 5 (completely true). The composite score for professional autonomy was determined by averaging the responses to these items ranging in a score between 1 (low level of professional autonomy) and 5 (high level of professional autonomy). In this study, the scale demonstrated a Cronbach’s alpha reliability coefficient of .78.

#### Self-epistemic autonomy

Assessment of the nursing student level of self-epistemic authority was based on a scale developed by Raviv et al. (2003) [9]. We adjusted from a teaching context to a nutrition context. The questionnaire consisted of nine items, including affirmations such as: “I possess extensive knowledge in nutrition”, and “I am capable of providing accurate responses to questions about nutrition”. Notably three of these items required reverse scoring, for example, “my understanding of nutrition does not surpass that of others”. Respondents were instructed to express the extent to which each statement best resonated with their experiences, using a 6-point Likert scale that ranges from 1 (strongly disagree) to 6 (strongly agree). The final self-epistemic autonomy score was calculated by averaging the responses to these nine items ranging between 1 (low level of self-epistemic autonomy) and 6 (high level of self-epistemic autonomy). The reliability of this scale, as indicated by Cronbach’s alpha, was 0.90 in this study.

#### Sense of meaning

This scale incorporated five questions extracted from the 24-item Calling and Vocation Questionnaire CVQ; Dik et al., (2012) [18], which were adapted to fit the current sample. These questions aimed to capture the nursing students’ perceptions of meaning and their sense of professional mission, with examples including, “I perceive the nursing profession as my calling”, “I am engaged in defining and understanding my mission within the nursing profession”, and “my career is a significant component of my life’s meaning”. Participants were required to assess how well each statement reflected their sentiments using a 4-point Likert scale, ranging from 1 (completely untrue) to 4 (completely true). The composite score for sense of meaning. Sense of meaning was calculated as the mean of these five items, the score ranging between 1 (low level of *sense of meaning*) and 4 (*sense of meaning*). The reliability of this measure, as indicated by Cronbach’s alpha, was 0.77 in this research.

### Procedure

The study commenced subsequent to obtaining approval from the ethics committee of the Tel Aviv-Yafo Academic College. Participants were approached in their classroom settings where they were provided with the research questionnaires alongside instructions requiring their signed consent for participation. These guidelines underscored the anonymity of the study and affirmed the participants’ right to withdraw at any point. The research team distributed the questionnaires to two distinct cohorts: one that had passed the clinical nutrition course and another yet to fulfill this requirement. Participants were instructed to fill out the questionnaire and ensure that no items were omitted.

### Handling missing data

To handle missing data, we performed multiple imputation using SPSS (version 23.0). The pattern and extent of missing data were first analyzed, revealing that 2.5% (150 missing data values and 5926 complete data values) of the data were missing at random across the four questionnaires (professional autonomy, professional authority, self-epistemic authority, sense of meaning).

We used the Fully Conditional Specification (FCS) method to generate five imputed datasets, which is within the recommended range for reliable imputation. The imputation model used predictive mean matching and included all variables from the primary analysis to ensure that the relationships among the data were preserved. Each imputation underwent 10 iterations to ensure convergence.

Diagnostic checks, including trace plots, were used to assess the convergence of the imputation process. Additionally, we compared the distributions of imputed values with observed values to ensure the quality of imputations. For the analysis, we used Rubin’s rules to combine parameter estimates and standard errors across the five imputed datasets.

*Statistical Analysis*, All statistical analyses were conducted using JASP version 0.18.2, which utilizes R version 4.3 as its underlying engine. Descriptive statistics of means and SDs were used to describe age and the four measures (professional identity, professional autonomy, self-epistemic authority, and *sense of meaning*) and Pearson correlation was used to describe the relationships between them. Frequencies and percentages were used to describe qualitative background characteristics (gender, religion, marital status, academic year, and whether they had participated in and completed a nutrition course). Independent samples t-tests were performed to compare the two cohort groups (had completed a nutrition course vs. had not) in the study’s four measures.

To investigate the mediation effects, we employed the Generalized Least Squares (GLS) method, implemented in JASP version 0.18.2. The GLS method was chosen due to its ability to handle potential issues of heteroscedasticity and autocorrelation in the data, providing more efficient and unbiased parameter estimates compared to Ordinary Least Squares (OLS) regression. GLS adjusts for variations in error variances and covariances, thereby offering a robust approach for estimating the relationships among the independent variable, mediator, and dependent variable. This method enhances the precision and reliability of the mediation analysis, ensuring that the effects are accurately captured, and the assumptions of the regression model are appropriately met.

To ensure the validity of our statistical analyses, we conducted assumption checks for Pearson correlations, t-tests, and mediation analysis. For Pearson correlations, we assessed linearity, homoscedasticity, and the absence of significant outliers using scatter plots and standardized residual plots. For t-tests, we tested normality using the Shapiro-Wilk test and inspected Q-Q plots, while Levene’s test was used to confirm homogeneity of variances for independent t-tests. In mediation analysis, we verified linearity and normality of residuals through residual plots and the Kolmogorov-Smirnov test, and then assessed homoscedasticity via standardized residual plots. Multicollinearity was checked using variance inflation factors (VIF), ensuring all VIF values were below 10. All assumptions were satisfactorily met, validating the robustness of our findings. Mediation model estimates and path coefficients were adjusted for gender and age.

## Results

The cohort comprised 98 participants, with a gender distribution of 73 females (74.5%) and 25 males (25.5%) and an age range of 18–42 years (*M* = 26.6, *SD* = 5.5). Among the 98 participants, 71 (72. 5%) were single, and 27 (27.6%) were married. A majority of 72 (72.5%) identified as Jewish Israelis, 21 (21.4%) as Muslim Israelis, with the remaining five affiliating with other religions. Table 1 shows the breakdown between the two cohorts (completed and did not attend a nutrition course) for these background variables and tests using chi-square for independent variables for any relationship with gender, marital status and religion and a t-test for independent samples for differences in age between the two cohorts. Groups were found to be significantly different in age and gender: 86% of nursing students who completed a nutrition course were female as opposed to 58.5% of students who did not attend a nutrition course. Students who completed a nutrition course were 2.5 years older than students who did not attend a nutrition course.

**Table 1:** Group differences in background variables

	Nutrition course				
	Completed course (*n* = 57)		Not attended course (*n* = 41)		
	*n*	*%*	*n*	*%*	*χ* ^*2*^
Gender (females)	49	86%	24	58.5%	
Marital status (single)	42	73.7%	29	70.7%	
Religion (Jewish)	42	73.7%	29	70.7%	
	*M*	*SD*	*M*	*SD*	*t*
5. Age	27.6	5.2	25.1	5.7	2.23*

* *p* < .05

The study variables showed a high level of professional identity (M = 3.30, Sd = 0.45; max score = 4), moderate levels of professional autonomy (M = 3.28, SD = 0.73; max score = 5) and self-epistemic authority (M = 3.81, SD = 1.06; max score = 6), and a high level of sense of meaning (M = 3.47, SD = 0.58; max score = 4). Pearson correlations were performed between study’s variables, as having non-zero correlations between the variables in a mediation model are a pre-requisite for mediation model testing (Table 2).

**Table 2:** Correlations between the research variables

	1		2		3		4		5	
1. Professional identity	—									
2. Professional autonomy	0.50	***	—							
3. Self-epistemic authority	0.57	***	0.56	***	—					
4. Sense of meaning	0.61	***	0.38	***	0.30	**	—			
5. Age	0.04		0.01		0.16		0.03		—	

* *p* < .05, ** *p* < .01, *** *p* < .001

As indicated in Table 2, there is no significant relationship between age and any of the variables under study. The relationships between self-epistemic authority and sense of meaning are characterized by a weak positive correlation, whereas the correlations between ‘professional identity’, ‘professional autonomy’, ‘self-epistemic authority’ and ‘sense of meaning’ were moderate positive correlations. A subsequent analysis involved examining the differences between two cohorts of nursing students: those who had completed a nutrition course (*n* = 57) and those who had not (*n* = 41), utilizing student’s t-test for independent samples. The outcomes of the t-test analyses, detailed in Table 3, reveal significantly elevated levels of Identity, Autonomy, and Epistemic Authority among nurses who participated in a nutrition course compared to those who did not. Conversely, no significant disparities in sense of meaning were observed between the two groups.

**Table 3:** T-test comparing the two nutrition course groups

	**After a nutrition course** **(n = 57)**						**No nutrition course** (n = 41)				t(96)
	*h*	*M*	*h*	*SD*	*h*		*M*	*h*	*SD*	h	
Professional identity		3.39		0.34			3.18		0.55		2.27*
Professional autonomy		3.45		0.66			3.03		0.76		2.90**
Self-epistemic authority		4.25		0.84			3.20		1.06		5.44***
Sense of meaning		3.51		0.49			3.42		0.68		0.72

* *p* < .05, ** *p* < .01, *** *p* < .001

### Mediation analysis

We then tested our hypothesis positing that professional autonomy and self-epistemic authority mediated the relationship between sense of meaning, and professional identity. This was accomplished through mediation analysis using Generalized Least Squares (GLS) standardized estimators. This mediation model clearly showed that professional autonomy did not mediate the relationship between sense of meaning, and professional identity Therefore, professional autonomy was excluded from the model (Table 4).

**Table 4:** Direct, indirect, and total effects of Epistemic Authority’s partial mediation of the relationship between Sense of Meaning and Professional Identity

Effect	Path	estimate	Std. error	Z
Direct	Sense of meaning → Professional identity	0.45	0.07	6.07***
Indirect	Sense of meaning → Self-epistemic authority → professional identity	0.09	0.04	2.37*
Indirect	Sense of meaning → professional autonomy → professional identity	0.04	0.03	1.33
Total	Sense of meaning → identity	0.59	0.08	7.47***

** *p* < .01, *** *p* < .001

Note: Delta method standard errors, GLS estimator, model adjusted for gender and age

**Diagram 1:**
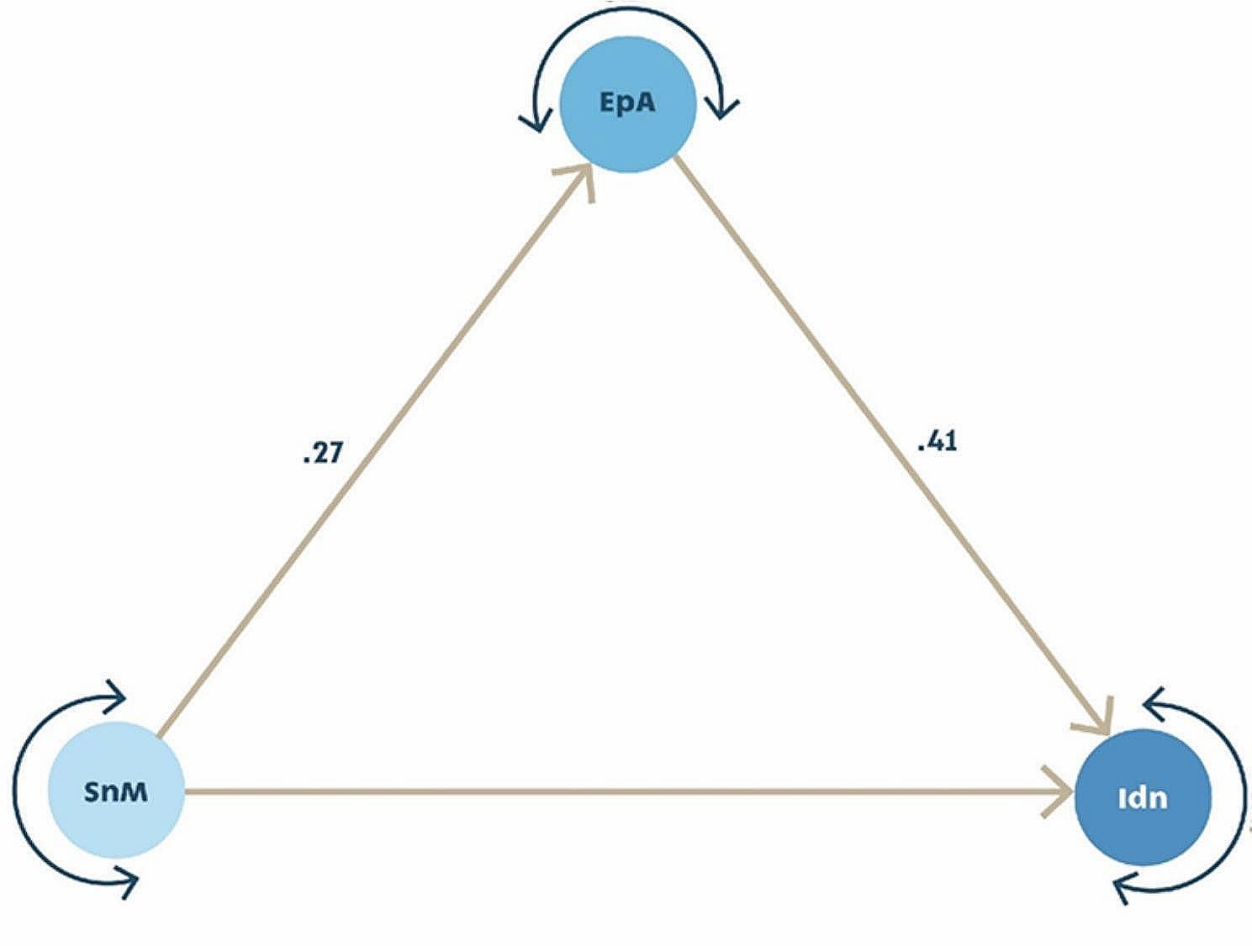
The mediation model Path coefficients of the mediation model with Epistemic Authority (EpA) partially mediating the relationship between sense of meaning (SnM) and Professional Identity (Idn)

The final mediation model revealed that self-epistemic authority partially mediated (accounting for 21.1% of the total effect) the relationship between sense of meaning and professional identity, as depicted in Table 5. Diagram 1 illustrates the path coefficients within the mediation model.

**Table 5:** The final mediation model: the relationship between a sense of meaning and professional identity

Effect	Path	estimate	Std. error	Z
Direct	Sense of meaning → professional identity	0.48	0.07	6.62***
Indirect	Sense of meaning → epistemic authority → professional identity	0.11	0.04	2.58**
Total	sense of meaning → identity	0.59	0.08	7.47***

** *p* < .01, *** *p* < .001

Note: Delta method standard errors, GLS estimator, model adjusted for gender and age

## Discussion

The present study reveals that nursing students who received a practical nutrition course showed a higher level of self-epistemic authority, professional autonomy, and professional identity than those in the control group who did not receive this course. However, the results show no significant disparity in the sense of professional meaning between the control and experimental cohorts. Furthermore, the study aims to assess the impact of the study variables on professional identity. Within the mediation model, professional autonomy was professional autonomy variable was identified as non-contributory and subsequently omitted from the model. However, in the mediated model, a sense of professional meaning emerges as one of the predictors of professional identity. The acquisition and application of clinical knowledge through appropriate training constitute fundamental aspects of nursing education, facilitating students’ capacity to retain and employ acquired knowledge in real-world scenarios [19]. The process of knowledge acquisition, coupled with the affirmation of one’s cognitive competencies, enhances one’s self-epistemic authority [9]. Studies show a significant relationship between self-epistemic authority and professional identity [14, 20], which aligns with the findings of the present study. Increasing self-epistemic authority and obtaining knowledge is crucial for nursing as it increases nursing care, fosters a sense of professional mission, and facilitates retention, thereby mitigating workforce attrition [21–23].

Interestingly, our results, derived by performing t-tests to compare the two cohorts under study, revealed that nursing students who completed a nutrition course had higher levels of professional identity, professional autonomy, and Epistemic authority compared to nursing students who did not attend a nutrition course. However, the two groups showed no difference in ‘sense of meaning’. This discrepancy could potentially be attributed to the variability in the educational stages or academic year of the nursing students participating in our study. Research published by Vabo and Fossum [24]. Indicates variations in the perceptions of sense of professional meaning and professional identity among students at varying junctures of their nursing education journey, producing sustainable development goals and widening participation [25–27].

When constructing a predictive model for professional identity based on the variables in our study, we observed that ‘sense of meaning’ and ‘self-epistemic authority’ played significant roles. However, professional autonomy had no significant contribution to the predictive model. Conversely, ‘sense of meaning’ significantly predicted professional identity. However, it should be considered whether this finding could be attributed to item similarity in the ‘sense of meaning’ and ‘professional identity’ questionnaires.

Despite these significant findings, the generalizability of the study results is influenced by several factors. The sample was drawn from a single academic institution, which may limit the applicability of the findings to nursing students from other institutions or regions. Additionally, the study utilized a convenience sample, which can introduce selection bias and may not represent the broader population of nursing students. The relatively small sample size further restricts the ability to generalize the findings, as it may not capture the full diversity of experiences and backgrounds present in the larger population of nursing students.

Moreover, the specific context of the nutrition course within the curriculum at the studied institution might differ from other programs, affecting the transferability of the results. Future research should aim to replicate this study with larger and more diverse samples across multiple institutions to enhance the external validity. Including students from different geographic locations, educational backgrounds, and varying stages in their nursing education would provide a more comprehensive understanding of how nutrition education impacts professional identity and related constructs.

Overall, while the study provides valuable insights into the relationship between nutrition education and professional identity among nursing students, caution should be exercised in generalizing these findings beyond the specific context and sample of this study. Further research is needed to confirm these results in broader and more varied populations.

The findings in the current study show that the acquisition of clinical knowledge increases nursing students’ self-epistemic authority, instills a sense of mission in the profession, and enhances their professional identity. Accordingly, we advocate for further scholarly inquiry into this domain positing that such endeavors could substantially contribute to fostering professional pride, curtailing attrition rates, and promote retention of nurses within this paramount profession.

## Data Availability

The data have the reserch participents name . However, The datasets used and/or analyzed in this study are available from the corresponding author upon reasonable request.
